# Bridging the gender gap in occupational wellbeing: a pilot intervention based on psychoeducation in judicial office for organized crime

**DOI:** 10.3389/fsoc.2026.1887312

**Published:** 2026-09-09

**Authors:** Valeria Saladino, Danilo Calaresi, Loriana Castellani, Stefano Eleuteri, Maria Giovanna Massari, Filippo Petruccelli, Irene Petruccelli, Valeria Verrastro, Fiorenza Giordano

**Affiliations:** 1 Department of Health Sciences, Magna Graecia University of Catanzaro, Catanzaro, Italy; 2 Faculty of Communication, Culture and Society, Università della Svizzera italiana, Lugano, Switzerland; 3 Department of Human, Social and Health Sciences, University of Cassino, Cassino, Italy; 4 Department of Human and Social Sciences, Mercatorum University, Rome, Italy; 5 Department of Human Sciences and Promotion of Quality of Life, San Raffaele Telematic University, Rome, Italy; 6 Institute for the Study of Psychotherapies, Rome, Italy

**Keywords:** emotional intelligence, gender-sensitive approach, occupational risk, psychoeducational intervention, workplace wellbeing

## Abstract

Occupational wellbeing is critical for organizational effectiveness in high-pressure public sectors, yet gender-neutral interventions often overlook nuanced identity dynamics. This exploratory pilot study evaluated a five-month, preventive, group-based psychoeducational intervention designed to moderate psychological risk and enhance emotional intelligence among 27 professionals in an Italian public security agency. Pre- and post-intervention evaluations assessed changes in emotional intelligence (WLEIS), psychological injury risk (PIRI), and job-related affective wellbeing (JAWS), alongside qualitative thematic analysis of focus group transcripts. Post-intervention trends suggested subtle, sex-disaggregated and age-moderated shifts: while female participants demonstrated moderate improvements in emotional intelligence and reductions in perceived psychological risk, male participants reported a minor reduction in self-assessed emotional skills and an increase in reported psychological strain. Age-disaggregated analyses revealed distinct cohort responses: while the 50 + group showed substantial gains in emotional intelligence and affective wellbeing, the 31–50 cohort exhibited a more critical self-appraisal of emotional skills. Despite limitations inherent to a small pilot sample, self-report reliance, and a single-arm non-experimental design, this work offers valuable preliminary insights into designing gender-sensitive, intersectional, and reproducible health promotion strategies within high-stakes public institutions.

## 1 Introduction

Occupational wellbeing plays a crucial role in shaping the psychological and functional health of employees and the overall performance of organizations (Bautista et al., 2023; Wei et al., 2025). Specifically, it refers to a state of balance between emotional, cognitive, and social factors that contribute to a worker’s capacity to engage meaningfully with their role (Doble and Santha, 2008; World Health Organization, 2019). When this balance is disrupted, especially in high-pressure, hierarchical environments, the risk of long-term emotional strain increases significantly (Zhao et al., 2022). Indeed, research in occupational health psychology has increasingly emphasized the importance of proactive interventions to sustain wellbeing and reduce work-related psychological risk (Belay et al., 2023).

A growing body of evidence has identified emotional intelligence (EI) as a key protective factor against occupational stress and burnout. Defined as the capacity to perceive, understand, use, and regulate emotions in oneself and others (Bru-Luna et al., 2021; Lam et al., 2022), EI contributes to more effective emotional regulation, interpersonal relationships, and adaptive responses to stressful situations (Ibrahim et al., 2024; Parker et al., 2021). Employees with higher levels of EI tend to interpret work-related stressors as manageable challenges rather than threats, use more functional coping strategies, and experience fewer symptoms of psychological distress (George et al., 2022; Geraci et al., 2023; Crudden et al., 2021; Zaghini et al., 2023). Importantly, emotional intelligence can be enhanced through training and psychoeducational interventions (Hubscher-Davidson and Lehr, 2021), making it a promising target for workplace programs aiming to foster resilience. It is also worth noting that the development and application of emotional skills do not occur in a vacuum. The ways in which employees perceive and regulate their emotions are frequently influenced by cultural and gender-based patterns of communication. For instance, while some might be socialized toward high emotional attunement, others may have been conditioned to prioritize emotional detachment.

Furthermore, literature on occupational health suggests that gender and age socialization may shape emotional expression, coping mechanisms, and help-seeking behaviors in organizations (Kim et al., 2020; Goel and Verma, 2021). Understanding whether psychoeducational interventions resonate differently across sex and age groups is therefore critical for designing targeted institutional health promotion.

While stress management programs are increasingly common in corporate and healthcare environments, empirical evaluations of tailored health promotion programs in confidential, anti-crime judicial agencies remain rare (Bidi et al., 2024; Christianson et al., 2023). Moreover, existing organizational interventions frequently treat participant cohorts as homogeneous, overlooking how sex, age, and organizational experience interact with intervention processes. To address this gap, the present exploratory pilot study evaluates a five-month psychoeducational program implemented within an Italian Judicial Office for Organized Crime. Rather than establishing definitive causal claims, this study adopts an exploratory, multi-parametric approach to investigate preliminary trends in emotional intelligence, psychological injury risk, and job-related affective wellbeing across sex and age groups, complemented by a rigorous qualitative investigation of participant dynamics.

## 2 Methods

### 2.1 Participants and procedures

The study was conducted in collaboration with an Italian Judicial Office for Organized Crime, a specialized public sector agency handling complex anti-crime investigations, intelligence analysis, and high-stakes legal proceedings. This specific population was selected due to the unique, chronic psychosocial stressors inherent to anti-crime judicial work, including high operational pressure, exposure to traumatic case material, and confidential working conditions. Despite these elevated risk factors, tailored, non-clinical occupational health promotion interventions designed for anti-crime legal and operational personnel remain relatively unstudied. A total of 27 full-time staff members voluntarily enrolled in the study (100% completion rate for pre- and post-assessments). Participation was uncompensated and strictly voluntary. Ethics approval was obtained from the local Institutional Ethics Committee (ISP-IRB-2024-4), and all participants provided written informed consent in accordance with the Declaration of Helsinki and Italian Association of Psychology (AIP) ethical guidelines.

No participants formally withdrew from the study (0% drop-out rate). However, session-level attendance varied between 6 and 10 members per subgroup due to unpredictable judicial deadlines and operational enforcement duties, resulting in an average session attendance rate of 85%.

The mean age of the sample was 44.2 years (SD = 8.6, range 28–59 years), divided into three age cohorts: <40 years (*n* = 9, 33.3%), 40–50 years (*n* = 11, 40.7%), and >50 years (*n* = 7, 25.9%). Average tenure in the judicial sector was 11.5 years (SD = 6.2, range 2–24 years), categorized into <10 years (*n* = 12, 44.4%) and ≥10 years (*n* = 15, 55.6%). Occupational roles comprised Administrative and Legal Support Staff (*n* = 16, 59.3%) and Operational and Investigative Support Officers (*n* = 11, 40.7%). Sex distribution included 15 female (55.6%) and 12 male (44.4%) participants.

### 2.2 Intervention design

To ensure methodological rigor and reproducibility, the intervention is detailed below in accordance:

1. Rationale and Goal: The program aimed to strengthen individual emotional competencies, foster psychological safety, and reduce work-related distress through a combined psychoeducational and experiential group framework.
2. Content and Delivery Modules: The intervention comprised 10 structured bi-weekly modules delivered over a 5-month period (total dosage = 40 contact hours). Each module consisted of a 2-h theoretical lecture followed by a 2-h experiential focus group (Table 1). Theoretical lectures utilized structured slide presentations, case vignettes, and psychoeducational literature. Experiential focus groups incorporated active learning modalities, including paired diaphragmatic breathing exercises, guided somatic relaxation, perspective-taking role-plays, reflective writing prompts, and structured peer feedback.
3. Facilitator Qualifications: Sessions were co-facilitated by two licensed clinical psychologists and psychotherapists with over 8 years of specialized experience in organizational health psychology and cognitive-behavioral group interventions.
4. Setting and Location: All sessions were conducted in person within confidential, soundproofed multi-purpose conference rooms located at the central headquarters of the Judicial Office for Organized Crime during working hours.
5. Group Allocation and Dosage: Participants were allocated to two parallel cohort groups for lectures (Cohort A: *n* = 13; Cohort B: *n* = 14) based on departmental scheduling availability to ensure non-disruption of agency duties. Within cohorts, participants were divided into stable focus sub-groups of 6–10 participants. Group membership remained fixed throughout the 5-month intervention to maintain trust and psychological safety.

**Table 1:** Demographic characteristics of the participants.

Demographic variable	Number of participants
Gender	
Female	22
Male	5
Age	
31–40 years	4
41–50 years	7
50 + years	16
Marital status	
Single	5
Married	11
Married with children	10
Not reported	1
Educational level	
Middle school diploma	1
High school diploma	11
University degree	8
Postgraduate education	7
Job position	
Judicial operator	6
Official	6
Expert clerk	5
Clerk	3
Employee	2
Judicial assistant	2
Director	1
IT assistant	1
Not reported	1
Years of service	
1–10 years	14
11–20 years	1
21–30 years	5
30 + years	7

The 10 integrated modules progressed systematically across three core phases:

- Phase 1: Foundation and Awareness (Modules 1–4): Covered work-related stress definitions, interpersonal communication styles, burnout prevention, and personal/formative need hierarchies. Lectures introduced theoretical concepts (e.g., Maslow’s hierarchy, stress models), while focus groups utilized stress-mapping and paired breathing to connect theory to personal emotional states.
- Phase 2: Competency and Skill Building (Modules 5–8): Targeted transversal soft skills (problem-solving), strategies for managing emotional stressors, effective communication, and leadership dynamics. Focus groups employed collaborative problem-solving, creative tasks to break habitual interaction patterns, and trust-building role-plays.
- Phase 3: Consolidation and Self-Efficacy (Modules 9–10): Focused on self-efficacy and overall emotional intelligence integration. Focus groups utilized projection-based reflection, personal success mapping, and resource sharing to translate learning into daily administrative and investigative routines.

### 2.3 Measures

To evaluate the impact of the intervention, the following standardized self-report instruments were administered pre- and post-intervention:

#### 2.3.1 Wong and Law Emotional Intelligence Scale

A 16-item self-report questionnaire developed by Wong and Law (2002) to assess emotional intelligence across four key dimensions: (1) Self-Emotional Appraisal (SEA), (2) Others’ Emotion Appraisal (OEA), (3) Use of Emotion (UOE), and (4) Regulation of Emotion (ROE). Each subscale consists of 4 items, and responses are given on a 7-point Likert-type scale ranging from 1 (strongly disagree) to 7 (strongly agree). The Italian version of the scale, validated by Iliceto and Fino (2017), has shown satisfactory reliability and factorial validity in workplace and organizational contexts, supporting its use for evaluating individual emotional competencies relevant to occupational wellbeing and interpersonal functioning.

#### 2.3.2 Psychological injury risk indicator

A 26-item self-report instrument designed to assess the risk of psychological injury due to occupational stress (Winwood et al., 2009). The Italian version, validated by Magnavita et al. (2015), has demonstrated excellent psychometric properties, including strong construct and structural validity, and high responsiveness. Items are rated on a 7-point Likert scale and contribute to four subscales: (A) Sleep Problems (6 items), (B) Recovery Failure (5 items), (C) Post-Traumatic Stress Symptoms (10 items), and (D) Chronic Fatigue (5 items). The factorial structure of the Italian version replicates the original Australian model and has been validated across diverse occupational groups (Magnavita et al., 2015).

#### 2.3.3 Job-related Affective Wellbeing Scale

The scale consists of 12 affective adjectives representing both positive and negative emotional states experienced in relation to one’s job (Warr, 1990). Respondents are asked to indicate, on a 5-point Likert scale ranging from 1 (never) to 5 (all of the time), how frequently their job made them feel each of the listed emotions in the past few weeks. The adjectives include: relaxed, worried, depressed, calm, contented, gloomy, optimistic, tense, enthusiastic, cheerful, miserable, and uneasy. The Italian version of the JAWS (Menghini et al., 2022) has been used in various occupational settings, showing adequate psychometric properties in terms of reliability and construct validity.

Quantitative assessment batteries (PIRI, WLEIS-I, JAWS) were administered in person via printed paper-and-pencil questionnaires immediately prior to Module 1 (T0) and within 1 week following Module 10 (T1). Questionnaires were completed individually in a private room and collected in sealed envelopes to ensure anonymity.

### 2.4 Quantitative data analysis

Quantitative data were analyzed using IBM SPSS Statistics Version 28. Given the exploratory pilot nature of the study and small sample size, data were analyzed using descriptive statistics pre- and post-intervention. Due to limited statistical power, emphasis is placed on descriptive trajectory shifts rather than confirmatory inferential assertions.

### 2.5 Qualitative methodology and thematic analysis

To rigorously investigate participant experiences, interpersonal shifts, and group dynamics during experiential focus groups, qualitative data were systematically collected and analyzed following Braun and Clarke (2006) six-phase thematic analysis framework. All focus group sessions were audio-recorded with participant consent and transcribed verbatim. Audio transcripts were supplemented by detailed field notes recorded by a non-participating observer who documented non-verbal behaviors, physical posture, group atmosphere, and emotional expressions.

Data analysis followed an inductive, data-driven approach. Two independent coders (clinical psychologists) thoroughly read the transcripts to establish familiarity, generated initial open codes, and organized codes into prospective sub-themes and overarching themes. Coding discrepancies were resolved through consensus meetings with a senior research supervisor.

## 3 Results

Exploratory quantitative analyses evaluated pre- to post-intervention shifts across emotional intelligence (WLEIS), psychological injury risk (PIRI), and job-related affective wellbeing (JAWS). Given the single-arm pilot design and small sample size (*N* = 27), descriptive metrics, mean changes, and subgroup comparisons across sex and age groups are presented to highlight preliminary observational trends rather than definitive causal effects.

### 3.1 Emotional intelligence (WLEIS-I)

Overall emotional intelligence scores showed a modest overall increase from pre- (*M* = 5.34) to post-intervention (*M* = 5.37).

Sex-disaggregated trends revealed divergent trajectories: female participants demonstrated an increase in self-reported emotional skills (T0: *M* = 5.34 vs. T1: *M* = 5.41), whereas male participants demonstrated a slight decrease in overall scores (T0: *M* = 5.31 vs. T1: *M* = 5.18).

**Figure 1:**
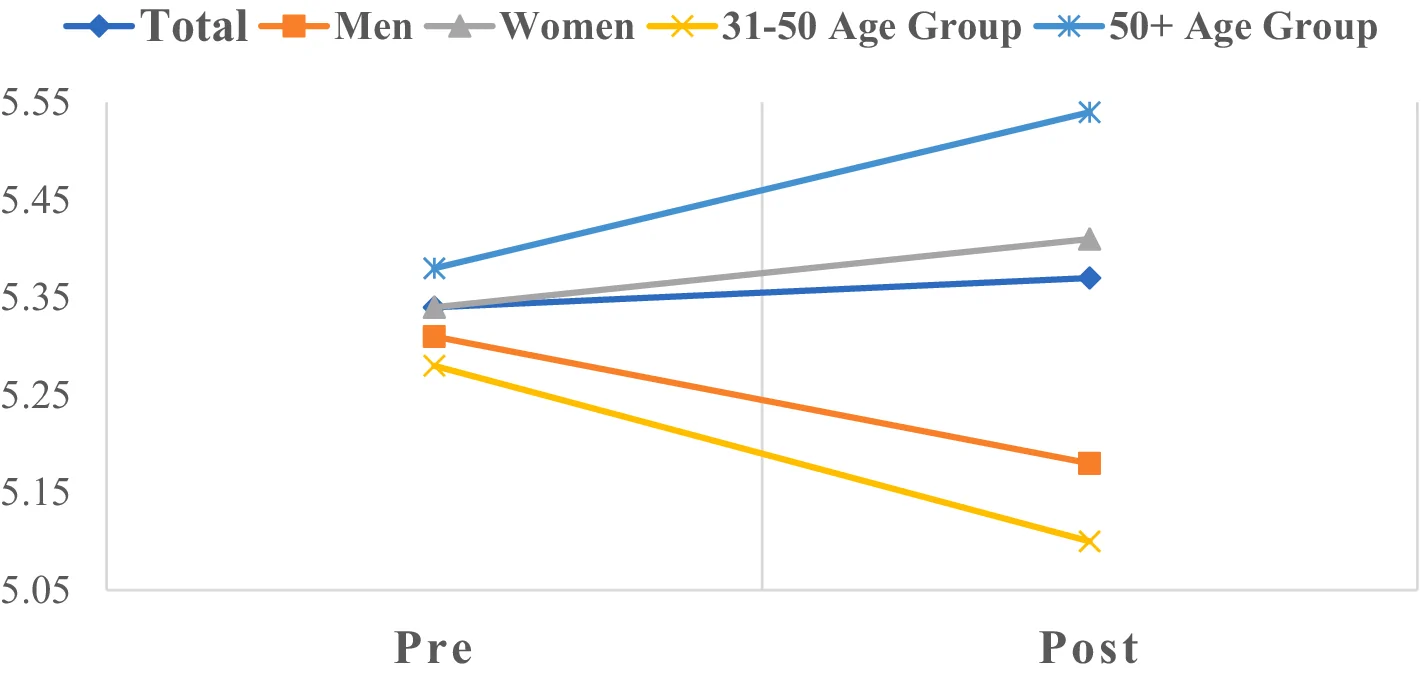
Comparison of pre-intervention and post-intervention scores on the Wong-Law Emotional Intelligence Scale (WLEIS-I).

Age-disaggregated trends showed that the 31–50 age cohort experienced a score decrease from baseline (T0: *M* = 5.28 vs. T1: *M* = 5.10), whereas the >50 age cohort showed an upward shift (T0: *M* = 5.38 vs. T1: *M* = 5.54) (Figure 1).

### 3.2 Psychological risk

Overall perceived psychological risk scores decreased from T0 (*M* = 1.82) to T1 (*M* = 1.50).

By sex, female participants reported a decrease in perceived risk scores (T0: *M* = 1.98 vs. T1: *M* = 1.30), while male participants reported an increase in reported psychological strain scores (T0: *M* = 1.11 vs. T1: *M* = 2.52).

**Figure 2:**
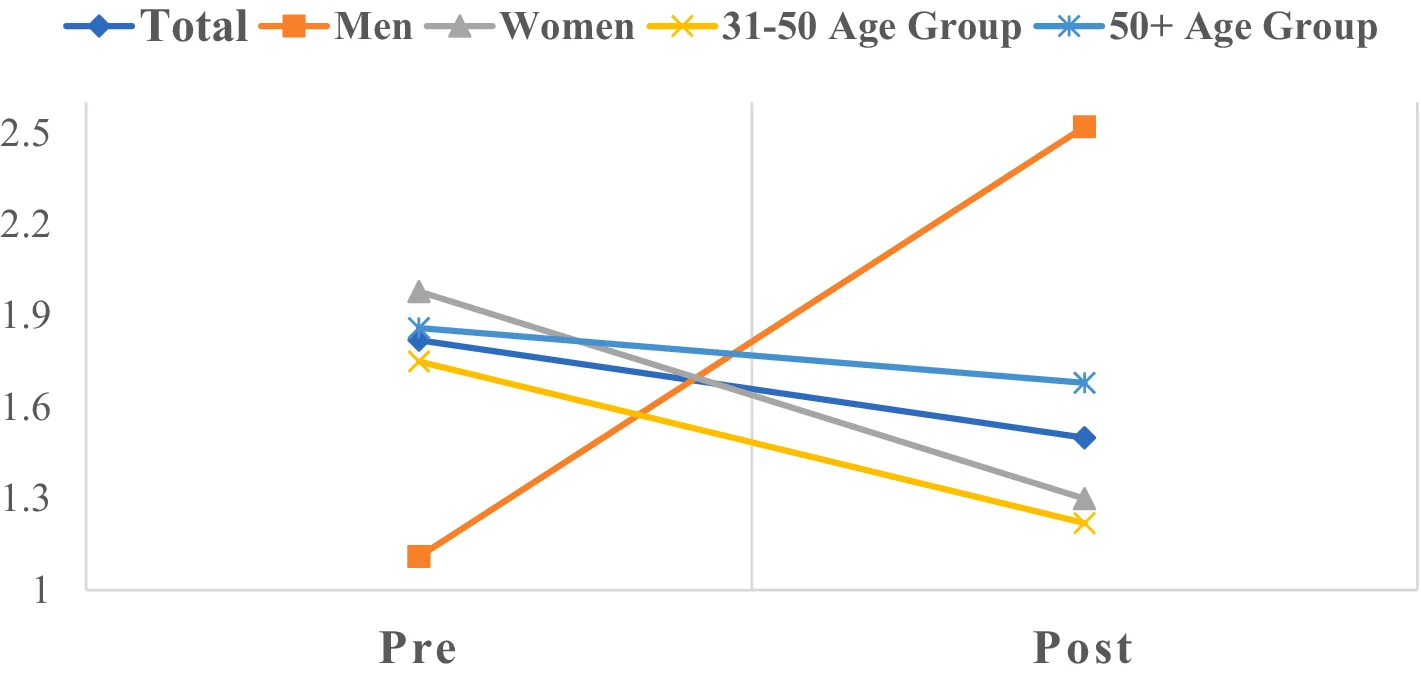
Comparison of pre-intervention and post-intervention scores on the Psychological Injury Risk Indicator (PIRI) Scale.

By age, the 31–50 cohort demonstrated a decrease in risk scores (T0: *M* = 1.75 vs. T1: *M* = 1.22), while the >50 cohort exhibited a flat trajectory (T0: *M* = 1.86 vs. T1: *M* = 1.68) (Figure 2).

### 3.3 Job-related affective wellbeing

Overall job-related affective wellbeing scores increased pre- (*M* = 3.20) to post-intervention (*M* = 3.43).

Both female (T0: *M* = 3.18 vs. T1: *M* = 3.39) and male participants (T0: *M* = 3.28 vs. T1: *M* = 3.61) reported positive score shifts.

**Figure 3:**
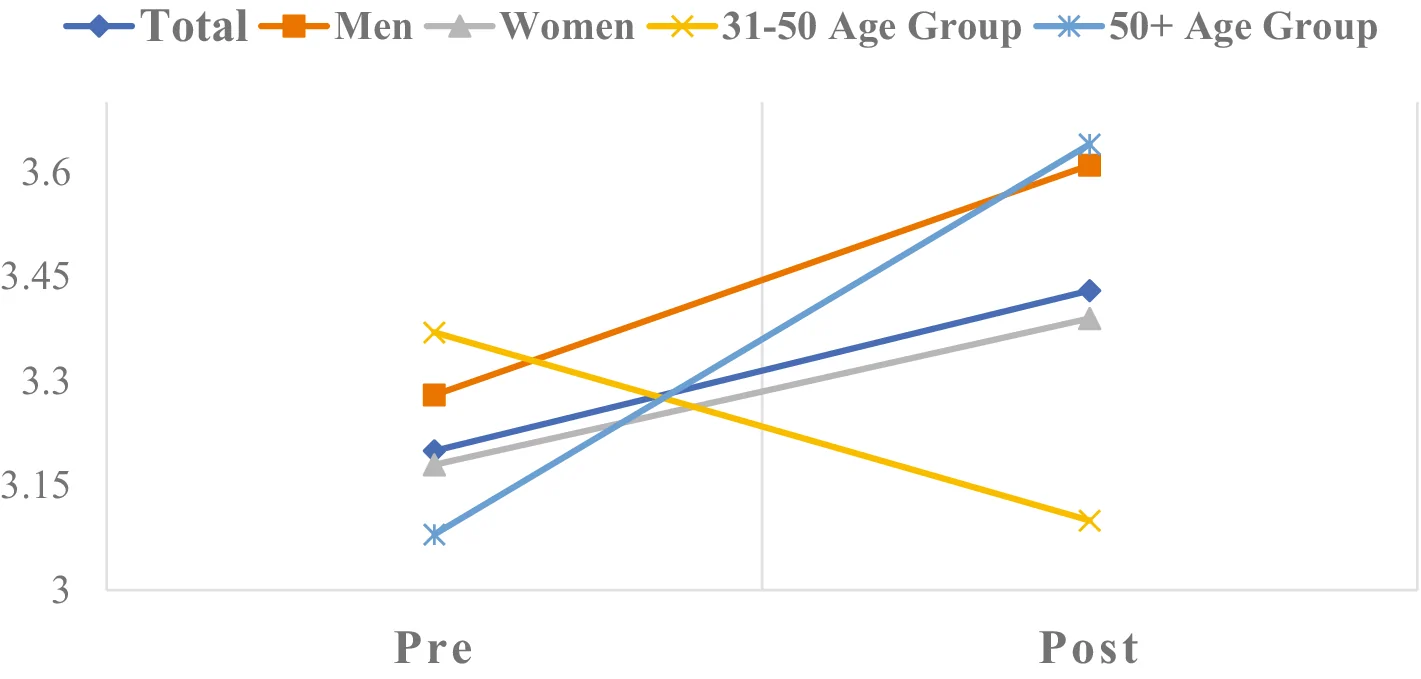
Comparison of pre-intervention and post-intervention scores on the Job-Related Affective Wellbeing Scale (JAWS).

By age, the >50 group exhibited an upward trajectory (T0: *M* = 3.08 vs. T1: *M* = 3.64), whereas the 31–50 age group showed a post-intervention decrease from their initial baseline (T0: *M* = 3.37 vs. T1: *M* = 3.10) (Figure 3).

### 3.4 Qualitative focus group findings

Inductive thematic analysis of focus group transcripts yielded four major qualitative themes illustrating the experiential trajectory of participants:

1. Initial Emotional Guardedness and Institutional Resistance: In early modules, participants, particularly male personnel and older staff, exhibited guarded body language, minimal verbal disclosure, and skepticism regarding the relevance of emotional self-appraisal in a judicial enforcement setting. Initial discussions were characterized by professional posturing and emotional detachment.
2. Dismantling Stoicism and Establishing Psychological Safety: As ground rules of confidentiality and non-judgment were reinforced, participants gradually transitioned from defensive postures to active engagement. The shared experiential exercises (e.g., paired breathing and stress mapping) served as catalysts for dismantling emotional suppression, creating a safe space for vulnerability.
3. Sex-Divergent Emotional Unmasking: Qualitative disclosures closely paralleled quantitative findings. Female participants used the focus group space primarily to de-escalate pre-existing workplace strain and build peer solidarity. Conversely, male participants engaged in “emotional unmasking,” acknowledging for the first time chronic fatigue, somatic strain, and moral burden that had previously been suppressed under institutional norms of stoicism.
4. Interpersonal Cohesion and Relational Clarity: By the final modules, participants reported enhanced mutual empathy, improved cross-departmental communication, and greater relational clarity, noting that skills learned in focus groups transferred to both daily administrative meetings and home environments.

## 4 Discussion

The findings of this pilot study suggest that a brief, group-based psychoeducational intervention can yield meaningful improvements in key indicators of occupational wellbeing, namely, emotional intelligence, psychological risk, and affective work-related wellbeing, in a high-demand organizational setting (Cabrita and Duarte, 2023; Choi et al., 2024; Daniels, 2000). These results align with a growing body of literature emphasizing the importance of emotional, cognitive, and interpersonal competencies in sustaining psychological health in the workplace (Doble and Santha, 2008; Bautista et al., 2023; Restrepo and Lemos, 2021; Shaholli et al., 2023). However, in light of the single-group design and exploratory nature of this evaluation, all findings must be interpreted with caution, avoiding strict causal attribution.

### 4.1 Alternative explanations for male psychological risk increases

Given the very small subgroup of male participants, any interpretation of gender-disaggregated trends must be treated as strictly exploratory and speculative. To rigorously contextualize the observed increase in male psychological risk scores (PIRI), several alternative theoretical and contextual explanations can be considered beyond a true clinical deterioration:

1. Sociocultural Norms and Willingness to Report Vulnerability: In high-stress, hierarchical public security and judicial environments, professional norms often emphasize emotional restraint and self-reliance, which can sometimes discourage the open reporting of vulnerability. The psychoeducational modules aimed to provide a safe framework to explore these professional coping styles. Consequently, changes observed in post-intervention assessments among some male participants could potentially reflect a greater willingness to acknowledge pre-existing chronic strain that had previously gone unreported, though this remains an exploratory hypothesis requiring further study in larger samples.
2. Measurement Sensitization and Testing Reactivity: Participating in 10 bi-weekly reflective modules focused on stress, burnout, and emotional regulation likely increased participants’ cognitive salience and self-monitoring regarding workplace stressors. This heightened sensitivity may have led male respondents to evaluate their daily working conditions with greater critical awareness at T1, resulting in elevated self-reported risk scores without representing a true clinical worsening.
3. Temporary Psychological Destabilization (“Worsening Before Improving”): Psychological and therapeutic literature recognizes that lowering defensive coping strategies during early stages of self-reflection frequently induces temporary emotional discomfort before new, adaptive coping mechanisms are fully consolidated. This transitional phase can transiently spike reported distress.
4. Exogenous Operational Context: The 5-month intervention took place within an active judicial office managing ongoing anti-crime operations. Uncontrolled external events, such as seasonal trial surges, high-profile case deadlines, or organizational staffing shifts, may have independently heightened acute strain among operational staff (who were predominantly male) during the post-assessment window.

### 4.2 The role of age

The integration of age-disaggregated analyses reveals a compelling and nuanced picture of how the intervention was received and processed across different life and career stages. Rather than yielding uniform effects, the program catalyzed distinct developmental and reflective trajectories between the 31–50 age group and the 50 + cohort, suggesting that professional maturity and career phase may influence how employees engage with resilience and emotional intelligence training.

These age-related dynamics underscore the limitation of treating the workforce as a monolithic entity. Interventions targeting emotional intelligence and psychological risk must account for generational and career-stage differences. Specifically, mid-career professionals (31–50) may require spaces that safely challenge existing coping mechanisms, accepting a temporary phase of vulnerability or critical self-doubt as a necessary step toward genuine resilience and lower psychological risk. Older workers (50+), on the other hand, benefit immensely from programs that validate their accumulated wisdom, translating it into immediate enhancements in daily affective wellbeing and emotional leadership.

### 4.3 Methodological limitations

Several methodological limitations must be acknowledged:

1. Heavy Reliance on Self-Report Measures: All quantitative outcomes (PIRI, WLEIS, JAWS) were captured via self-report instruments, which are inherently vulnerable to social desirability, retrospective recall bias, and common method variance. Future research should incorporate objective physiological indicators (e.g., heart rate variability, cortisol) or behavioral performance metrics.
2. Single-Arm Exploratory Design: The study lacked a randomized or non-equivalent control group. Without a comparison control condition, observed shifts cannot be definitively attributed to specific intervention components versus non-specific group effects or historical confounding factors.
3. Sample Size and Selection Constraints: The sample size (*N* = 27) was small and drawn from a single specialized institution, restricting statistical power and ecological generalizability to broader public sectors.
4. Group Attrition and Dosage Variations: While total completion was 100%, attendance in optional experiential focus group sessions fluctuated (ranging from 10 to 6 regular members in specific subgroups), creating potential variations in intervention dosage.
5. Absence of Long-Term Follow-up: Assessments were limited to immediate post-intervention (T1). Long-term longitudinal follow-up (e.g., 6 or 12 months) is needed to determine the persistence of observed affective improvements.

## 5 Conclusion

This exploratory pilot study highlights the feasibility and potential benefits of implementing structured psychoeducational and experiential interventions in high-demand judicial agencies. The findings emphasize that occupational wellbeing initiatives do not produce uniform effects across diverse staff cohorts. Sex, age, and organizational tenure moderate how stress awareness and emotional training are absorbed. While elevated risk reporting among male personnel warrants careful monitoring, it may tentatively signal a reflective diagnostic phase wherein institutional stoicism is gradually unmasked, though this hypothesis requires verification in larger cohorts. Future health promotion programs in public security and legal institutions must adopt multi-parametric, gender-sensitive, and reproducible designs to support long-term workforce resilience.

## Data Availability

The original contributions presented in the study are included in the article/supplementary material, further inquiries can be directed to the corresponding author.
